# Association between statin use and 30-day mortality among patients with sepsis-associated encephalopathy: a retrospective cohort study

**DOI:** 10.3389/fneur.2024.1371314

**Published:** 2024-12-27

**Authors:** Junwei Chen, Junde Chen, Lihong Li

**Affiliations:** ^1^The School of Clinical Medicine, Fujian Medical University, Fujian, China; ^2^Department of Emergency, The Affiliated Hospital of Putian University, Putian, China

**Keywords:** statins, sepsis-associated encephalopathy, 30-day mortality, MIMIC-IV, retrospective cohort

## Abstract

**Aim:**

Sepsis-associated encephalopathy (SAE) is a common and serious complication of sepsis with poor prognosis. Statin was used in SAE patients, whereas its effects on these patients remain unknown. This study is aimed at investigating the impact of statins on the 30-day mortality of patients with SAE.

**Methods:**

In this retrospective cohort study, data from SAE patients were extracted from the Medical Information Mart for Intensive Care IV (MIMIC-IV). Statins include atorvastatin, pravastatin, rosuvastatin, and simvastatin. The outcome was 30-day mortality of SAE patients starting 24 h after the first intensive care unit (ICU) admission and at the first time after hospitalization. Potential covariates (sociodemographic characteristics, vital signs, score indexes, laboratory parameters, comorbidities, and treatment intervention methods) were selected using univariate Cox proportional hazard analysis. Associations between statin use and statin type and 30-day mortality were explored using univariate and multivariate Cox proportional hazard models with hazard ratios (HRs) and 95% confidence intervals (CIs). Associations were further explored in different age groups, sex, sequential organ failure assessment (SOFA), simplified acute physiology score II (SAPS II), and systemic inflammatory response syndrome (SIRS) populations.

**Results:**

A total of 2,729 SAE patients were included in the study, and 786 (28.8%) died within 30 days. Statin use was associated with lower odds of 30-day mortality (HR = 0.77, 95%CI: 0.66–0.90) in all SAE patients. Patients who took simvastatin treatments were associated with lower odds of 30-day mortality (HR = 0.58, 95%CI: 0.43–0.78). Rosuvastatin treatments had a higher 30-day mortality risk (HR = 1.88, 95%CI: 1.29–2.75). Statin use was also associated with lower 30-day mortality among patients of different ages, sex, sequential organ failure assessment (SOFA), SAPS II, and SIRS.

**Conclusion:**

Patients who were treated with simvastatin were associated with lower odds of 30-day mortality in SAE patients. Caution should be paid to statin use in SAE patients, particularly in patients treated with rosuvastatin or pravastatin.

## Introduction

Sepsis-associated encephalopathy (SAE), a common complication of sepsis, is characterized by cognitive dysfunction, changes in mental status, and delirium (1, 2). The prevalence of SAE in septic patients ranges from 8 to 70%, depending on the criteria used to define SAE (2, 3). SAE denotes alterations in consciousness that lack direct proof of infection in the central nervous system. Compared with sepsis patients without SAE, SAE patients are associated with elevated mortality, increased utilization of intensive care unit (ICU) resources, and prolonged hospitalization (4). Furthermore, mortality increases with the severity of SAE and is almost 70% in severe SAE patients (5). Identifying modifiable factors that affect mortality in SAE is important for improving the prognosis of SAE and lessening the disease burden.

An essential aspect of SAE treatment is the appropriate management of systemic infections, sepsis, and systemic inflammatory response syndrome (6). Plausible mechanisms of SAE include neuroinflammation, disruption of the blood–brain barrier, alteration of cerebral microcirculation, and oxidative stress (7). Owing to the favorable anti-inflammatory, antioxidative, and immunomodulatory effects of statins, they have been investigated as a cost-effective and feasible alternative or adjunctive host-directed therapy for infectious diseases (8). In animal models, atorvastatin exerted anti-inflammatory and antioxidant effects in the cerebral cortex (9). Simvastatin, by exerting a neuroprotective role, effectively mitigates long-term cognitive impairments in rats that survived sepsis (10). Lee et al. (11) reported the administration of either atorvastatin or simvastatin before sepsis showed an association with enhanced survival rates at the 30-day survival. Rosuvastatin also was protective against SAE (12). In another study, statin use was associated with lower odds of 30-day and 90-day mortality in patients hospitalized with sepsis (13). In early sepsis, statin use in the ICU was associated with reduced delirium, with delirium being a performance indicator of SAE (14).

Statins have been recommended as a treatment for SAE; however, the effect of statins on the prognosis of SAE remains unexplored. We aimed to investigate the relationship between statin use and 30-day mortality in SAE patients and further explore the effects of different types of statins.

## Methods

### Data source

Data of SAE patients in this retrospective cohort study were extracted from the Medical Information Mart for Intensive Care IV (MIMIC-IV). Health-related data from the Beth Israel Deaconess Medical Center were included from the MIMIC-IV (2008–2019). The Institutional Review Board of both the Massachusetts Institute of Technology and Beth Israel Deaconess Medical Center approved the study (approval number: #2001P001699). No informed consent was required for the utilization of unidentified health data of the patients. The author completed the Collaborative Institutional Training Initiative course and passed both the “Conflicts of Interest” and “Data or Specimens Only Research” examinations. The hospital Ethics Committee waived the requirement for ethical approval for this study.

### Study population

Patients were included as follows: (1) diagnosed with SAE, (2) 18–89 years old, (3) complete information on statin use and statin types, and (4) ICU hospitalization ≥24 h. SAE was defined as a Glasgow Coma Scale (GCS) score < 15 on the first day of ICU admission or diagnosed delirium according to the International Classification of Disease-9 (ICD-9) code (2,930 and 2,931) or ICD-10 code (F05) in sepsis patients (15). Participants with a history of acute brain injury (e.g., meningitis, encephalitis, status epilepticus, traumatic brain injury, or stroke) and alcohol abuse were excluded. These exclusions were made to reduce diagnostic ambiguity and ensure the homogeneity of the study population.

### Statin use

The prescription drug file was used to identify medication exposure based on the generic and brand names. Statins include atorvastatin, pravastatin, rosuvastatin, and simvastatin. Standardized statin doses are shown in Supplementary Table S1.

### Covariates

The covariates were extracted based on socio-demographic characteristics (age, sex, ethnicity, insurance, and marital status), vital signs (heart rate, temperature, respiratory rate, and urine output), score indices [Charlson comorbidity index (CCI), sequential organ failure assessment (SOFA), simplified acute physiology score II (SAPS II), and systemic inflammatory response syndrome (SIRS)], laboratory parameters [white blood cell (WBC), platelet, hemoglobin, red blood cell distribution width-coefficient of variation (RDW-CV), serum creatinine, anion gap, and magnesium], comorbidities [acute kidney injury (AKI), respiratory failure, and cardiogenic shock], and intervention methods (machine ventilation, renal replacement therapy (RRT), midazolam, and propofol). CCI is the gold standard tool in clinical research as a prognostic index to predict mortality (16). The SOFA score was used to measure organ dysfunction in patients admitted to the ICU (17). The SAPS II was also used to predict in-hospital mortality. The data of physiological and laboratory indices were extracted from the first measurement taken during the period of ICU admission to ICU stay of less than 24 h. AKI was defined according to the Kidney Disease Improving Global Outcomes criteria (18). Respiratory failure was determined using the International Classification of Disease-9 (ICD-9) codes “51,881,” “51,883,” and “51,884” and the ICD-10 code “J96.” Cardiogenic shock was defined using ICD-9 code “78,551” and ICD-10 code “R570.” SAE phenotypes include ischemic–hypoxic, metabolic, mixed, and unclassified phenotypes (19).

### Outcome

The outcome was 30-day mortality. The database is followed by information in the electronic medical charts and hospital department records, or by making contact with the patients, their family members, their attending healthcare workers, or family physicians on the phone. The follow-up started 24 h after the first ICU admission and the first time after hospitalization and ended when patients died or 30 days after admission to the ICU.

### Statistical analysis

Continuous variables are presented as means and standard deviations (SD) for normally distributed data and as medians and interquartile ranges for non-normally distributed data. Categorical variables are presented as numbers and percentages (%). For continuous data, *t*-tests were used for normally distributed data, and Mann–Whitney rank-sum tests were conducted for non-normally distributed data. Categorical data were compared using the chi-squared tests. The univariate Cox proportional hazards model was used to select potential covariates. Covariates associated with 30-day mortality (*p* < 0.05) were included in the adjustment of the multivariable Cox model. Multivariate Cox proportional hazard models with hazard ratios (HR) and 95% confidence intervals (CI) were used to explore the association between statin use and statin type and 30-day mortality in SAE. We conducted a secondary analysis using propensity score matching (PSM) to reduce the effects of confounding factors and the likelihood of selection bias. Propensity matching was performed using a nearest-neighbor algorithm with 1:1 matching without replacement and a caliper distance of less than 0.1 (Supplementary Figures S1, S2). Absolute risk reduction (ARR) and the number needed to treat (NNT) have been used to estimate the potential benefit of statin therapy. Variables with a missing rate ≤ 20% underwent multiple imputations. Subsequently, a sensitivity analysis was conducted to compare variables before and after imputation. Assuming that the data are missing completely at random (MCAR), missing values can be predicted and interpolated from observed values. Subgroup analyses, which were stratified by age, sex, SOFA, SAPS II, and SIRS, were further investigated to determine the associations between statin use and statin types and 30-day mortality. Kaplan–Meier survival curves were constructed to compare 30-day mortality between statin use and non-statin use groups and between different types of statin use groups. Statistical significance was set at *p* < 0.05. Navicat Premium 16 (version 16.0.11) was used to extract data from the database. All analyses were performed using SAS 9.4 (SAS Institute Inc., Cary, NC, United States). The tables of statins used in different categories were plotted using GraphPad Prism 8.0.1.

## Results

### Characteristics of SAE patients

**Figure 1:**
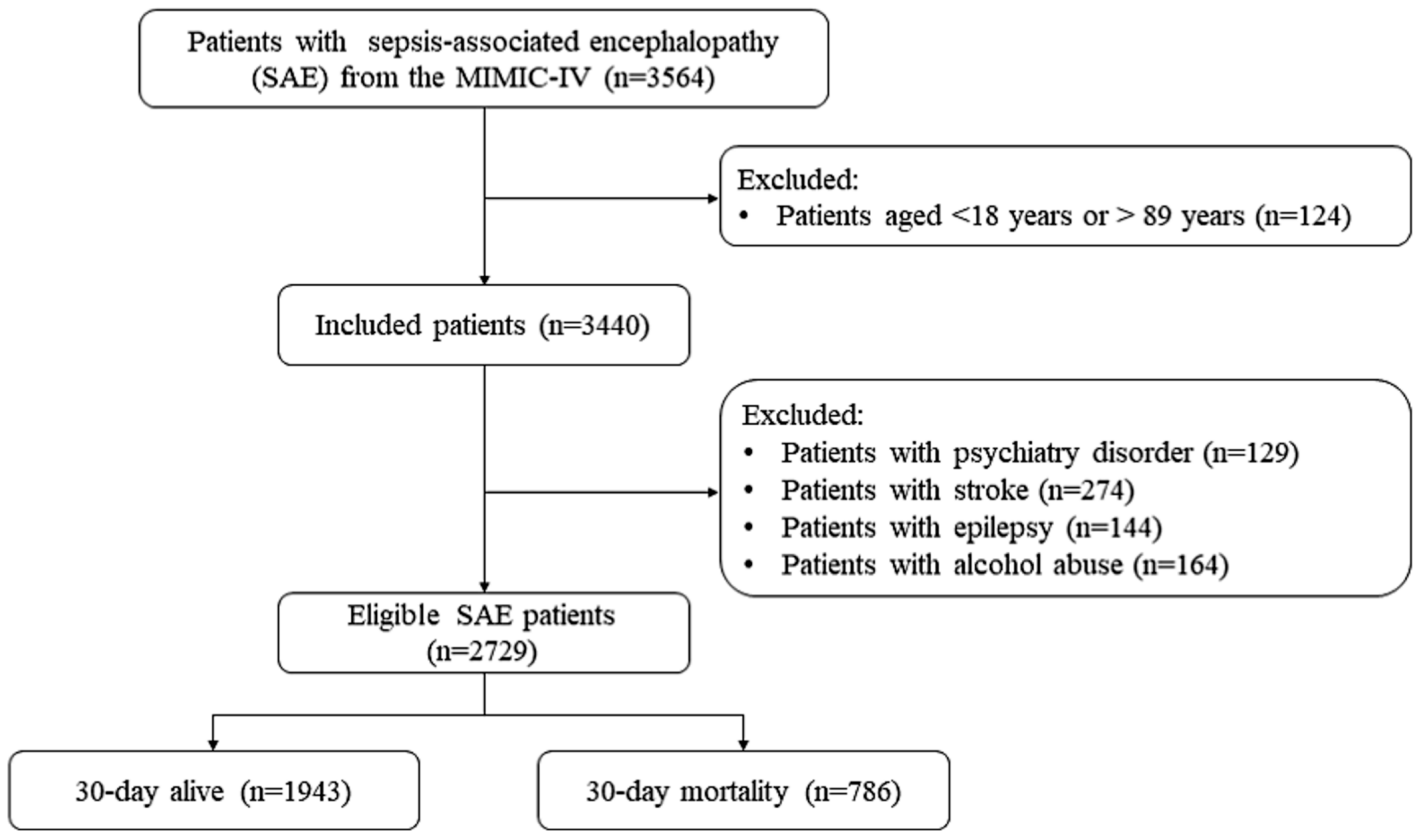
Flowchart of included sepsis-associated encephalopathy patients.

Figure 1 shows the SAE patient screening process. No significant differences were observed before and after data interpolation for the missing values (Supplementary Tables S2, S3). A total of 3,564 patients with SAE were initially included. Individuals aged <18 and > 89 years were excluded (*n* = 124). Next were patients with psychiatric disorders (*n* = 129), stroke (*n* = 124), epilepsy (*n* = 144), or alcohol abuse (*n* = 164). Finally, 2,729 patients with SAE were eligible. Supplementary Table S1 shows that no statistical difference was found before and after interpolation of the missing variables.

**Figure 2:**
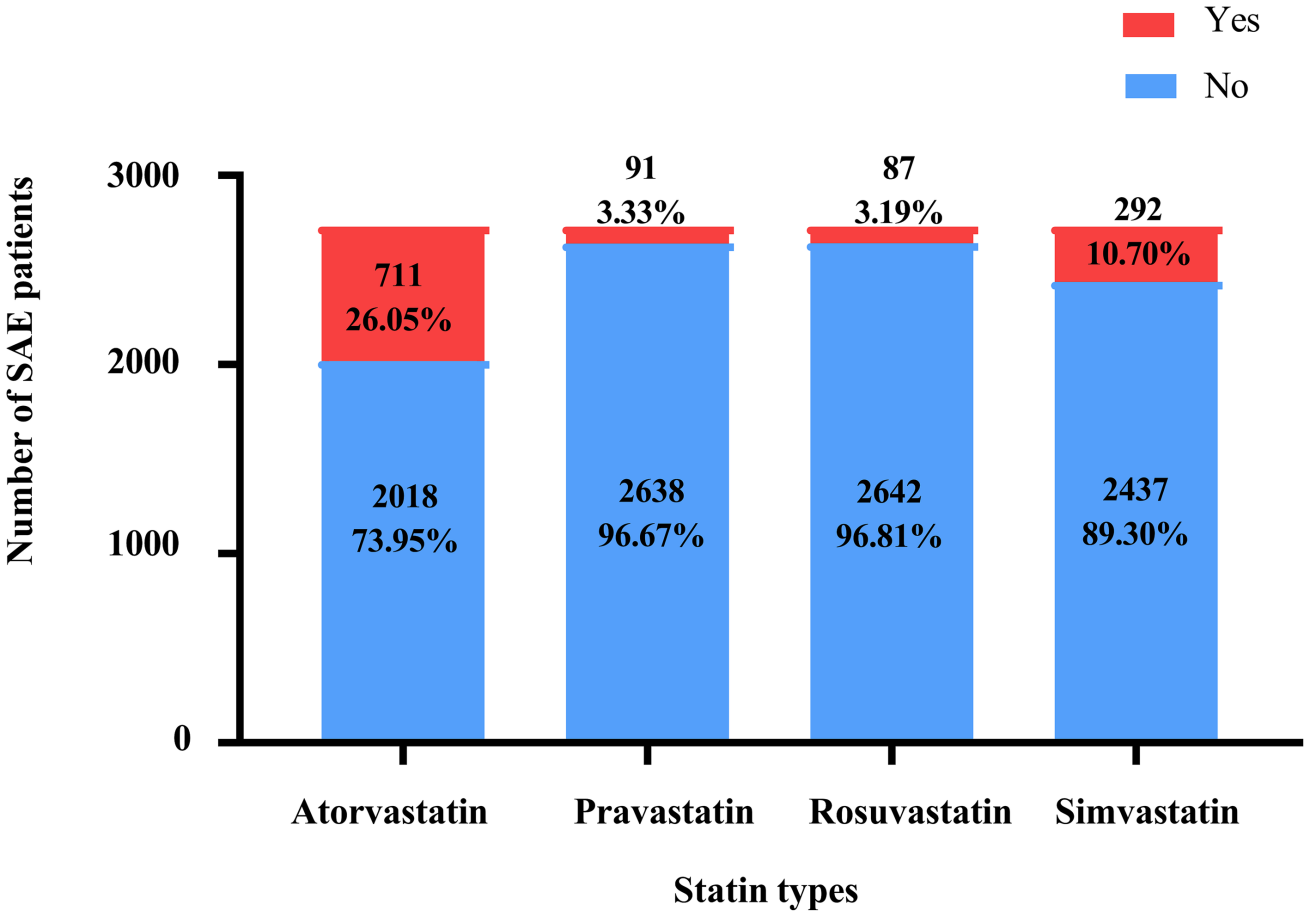
The proportion of different types of statins in SAE patients.

Table 1 presents the characteristics of the eligible SAE patients. Among 3,564 SAE patients, 1,133 received statin treatment, of which 664, 82, 81, and 258 were treated with atorvastatin, pravastatin, rosuvastatin, and simvastatin, respectively. And 48 patients were treated with a combination of statins. The mean age of all SAE patients was 64.84 ± 15.18 years old, and 57.93% (*n* = 1,581) were male. Among them, 786 (28.80%) died within 30 days. There were statistically significant differences between the two groups in age, insurance, AKI, respiratory failure, cardiogenic shock, urine output, respiratory rate, temperature, GCS, CCI, SOFA, SAPSII, SIRS, WBC, platelet, hemoglobin, RDW-CV, serum creatinine, anion gap, magnesium, machine ventilation, RRT, midazolam, propofol, type of statins, and SAE phenotypes (all *p* < 0.05). The characteristics of the eligible patients with SAE after PSM are presented in Supplementary Table S4. The use status of the different statins is shown in Figure 2.

**Table 1:** Characteristics of patients with sepsis-associated encephalopathy.

Variables	Total (*n* = 2,729)	30-day mortality		Statistics	*P*
		No (*n* = 1,943)	Yes (*n* = 786)		
Age, years, Mean ± SD	64.84 ± 15.18	63.21 ± 15.50	68.89 ± 13.54	t = −9.51	<0.001
Sex, n (%)				χ^2^ = 2.471	0.116
Female	1,148 (42.07)	799 (41.12)	349 (44.40)		
Male	1,581 (57.93)	1,144 (58.88)	437 (55.60)		
Ethnicity, n (%)				χ^2^ = 7.404	0.060
White	1787 (65.48)	1,273 (65.52)	514 (65.39)		
Black	253 (9.27)	189 (9.73)	64 (8.14)		
Other	293 (10.74)	218 (11.22)	75 (9.54)		
Unknown	396 (14.51)	263 (13.54)	133 (16.92)		
Insurance, n (%)				χ^2^ = 30.564	<0.001
Medicaid	197 (7.22)	153 (7.87)	44 (5.60)		
Medicare	1,323 (48.48)	877 (45.14)	446 (56.74)		
Other	1,209 (44.30)	913 (46.99)	296 (37.66)		
Marital status, n (%)				χ^2^ = 4.283	0.117
Married	1,191 (43.64)	840 (43.23)	351 (44.66)		
Unmarried	1,250 (45.80)	910 (46.83)	340 (43.26)		
Unknown	288 (10.55)	193 (9.93)	95 (12.09)		
AKI, n (%)				χ^2^ = 13.762	<0.001
No	911 (33.38)	690 (35.51)	221 (28.12)		
Yes	1818 (66.62)	1,253 (64.49)	565 (71.88)		
Respiratory failure, n (%)				χ^2^ = 50.181	<0.001
No	1,105 (40.49)	869 (44.72)	236 (30.03)		
Yes	1,624 (59.51)	1,074 (55.28)	550 (69.97)		
Cardiogenic shock, n (%)				χ^2^ = 10.619	0.001
No	2,627 (96.26)	1885 (97.01)	742 (94.40)		
Yes	102 (3.74)	58 (2.99)	44 (5.60)		
Urine output, mL, M (Q_1_, Q_3_)	1300.00 (750.00, 2085.00)	1390.00 (875.00, 2206.00)	1050.00 (524.00, 1800.00)	Z = −8.409	<0.001
Heart rate, bpm, Mean ± SD	93.99 ± 22.18	93.79 ± 22.54	94.51 ± 21.25	t = −0.77	0.440
Respiratory rate, insp/min, Mean ± SD	21.07 ± 6.85	20.81 ± 6.74	21.71 ± 7.09	t = −3.11	0.002
Temperature, Deg.C, Mean ± SD	36.76 ± 0.96	36.81 ± 0.96	36.64 ± 0.94	t = 4.21	<0.001
GCS, M (Q_1_, Q_3_)	13.00 (9.00, 14.00)	13.00 (9.00, 14.00)	13.00 (9.00, 14.00)	Z = −2.878	0.004
Charlson comorbidity index, M (Q_1_, Q_3_)	3.00 (2.00, 5.00)	3.00 (1.00, 5.00)	4.00 (3.00, 6.00)	Z = 11.395	<0.001
SOFA, M (Q_1_, Q_3_)	8.00 (5.00, 11.00)	7.00 (5.00, 11.00)	9.00 (6.00, 13.00)	Z = 8.721	<0.001
SAPS II, M (Q_1_, Q_3_)	44.00 (35.00, 56.00)	42.00 (34.00, 54.00)	50.00 (40.00, 60.00)	Z = 10.629	<0.001
SIRS,Mean ± SD	2.90 ± 0.88	2.87 ± 0.90	2.96 ± 0.82	t = −2.45	0.015
WBC, k/μL, M (Q_1_, Q_3_)	12.00 (8.30, 17.00)	11.80 (8.10, 16.40)	12.60 (8.70, 18.40)	Z = 3.479	<0.001
Platelet, k/μL, M (Q_1_, Q_3_)	185.00 (124.00, 258.00)	191.00 (131.00, 259.00)	171.50 (106.00, 249.00)	Z = −4.132	<0.001
Hemoglobin, g/dL, Mean ± SD	10.45 ± 2.37	10.60 ± 2.40	10.10 ± 2.27	t = 4.97	<0.001
RDW-CV, %, Mean ± SD	15.73 ± 2.66	15.34 ± 2.39	16.69 ± 3.03	t = −11.13	<0.001
Serum Creatinine, mg/dL, M (Q_1_, Q_3_)	1.20 (0.80, 2.00)	1.10 (0.80, 1.80)	1.40 (0.90, 2.40)	Z = 6.120	<0.001
Anion gap, meq/L, Mean ± SD	15.85 ± 4.89	15.59 ± 4.74	16.50 ± 5.17	t = −4.26	<0.001
Magnesium, mg/dL, Mean ± SD	2.03 ± 0.49	2.01 ± 0.52	2.06 ± 0.42	t = −2.12	0.034
Machine ventilation, n (%)				χ^2^ = 5.354	0.021
No	519 (19.02)	391 (20.12)	128 (16.28)		
Yes	2,210 (80.98)	1,552 (79.88)	658 (83.72)		
Vasopressors, n (%)				χ^2^ = 1.827	0.176
No	1,465 (53.68)	1,059 (54.50)	406 (51.65)		
Yes	1,264 (46.32)	884 (45.50)	380 (48.35)		
RRT, n (%)				χ^2^ = 10.002	0.002
No	2,493 (91.35)	1796 (92.43)	697 (88.68)		
Yes	236 (8.65)	147 (7.57)	89 (11.32)		
Midazolam, n (%)				χ^2^ = 5.041	0.025
No	2092 (76.66)	1,467 (75.50)	625 (79.52)		
Yes	637 (23.34)	476 (24.50)	161 (20.48)		
Propofol, n (%)				χ^2^ = 45.516	<0.001
No	1,534 (56.21)	1,013 (52.14)	521 (66.28)		
Yes	1,195 (43.79)	930 (47.86)	265 (33.72)		
Dexmedetomidine, n (%)				χ^2^ = 2.827	0.093
No	2,568 (94.10)	1819 (93.62)	749 (95.29)		
Yes	161 (5.90)	124 (6.38)	37 (4.71)		
Statins use, n (%)				χ^2^ = 3.668	0.055
No	1,596 (58.48)	1,114 (57.33)	482 (61.32)		
Yes	1,133 (41.52)	829 (42.67)	304 (38.68)		
Type of statins, n (%)				χ^2^ = 20.058	0.001
No use	1,596 (58.48)	1,114 (57.33)	482 (61.32)		
Atorvastatin	664 (24.33)	479 (24.65)	185 (23.54)		
Pravastatin	82 (3.00)	60 (3.09)	22 (2.80)		
Rosuvastatin	81 (2.97)	47 (2.42)	34 (4.33)		
Simvastatin	258 (9.45)	208 (10.71)	50 (6.36)		
More than one type	48 (1.76)	35 (1.80)	13 (1.65)		
SAE phenotypes, n (%)				χ^2^ = 61.799	<0.001
Ischemic–hypoxic SAE	605 (22.17)	440 (22.65)	165 (20.99)		
Metabolic SAE	449 (16.45)	276 (14.20)	173 (22.01)		
Mixed SAE	256 (9.38)	147 (7.57)	109 (13.87)		
Unclassified SAE	1,419 (52.00)	1,080 (55.58)	339 (43.13)		
Survival time, day, M (Q_1_, Q_3_)	30.00 (21.30, 30.00)	30.00 (30.00, 30.00)	11.23 (7.25, 17.30)	Z = −51.245	<0.001

t, *t*-test; Z, rank sum test; χ^2^, chi-square test; SD, standard deviation; M, median; Q_1_, 1st Quartile; Q_3_, 3rd Quartile. AKI, acute kidney injury; GCS, Glasgow Coma Scale; SOFA, sequential organ failure assessment; SAPS II, simplified acute physiology score II; SIRS, systemic inflammatory response syndrome; WBC, white blood cell; RDW, red blood cell distribution width; RRT, renal replacement therapy; SAE, sepsis-associated encephalopathy.

### Associations of statin use with 30-day mortality in all SAE patients

**Figure 3:**
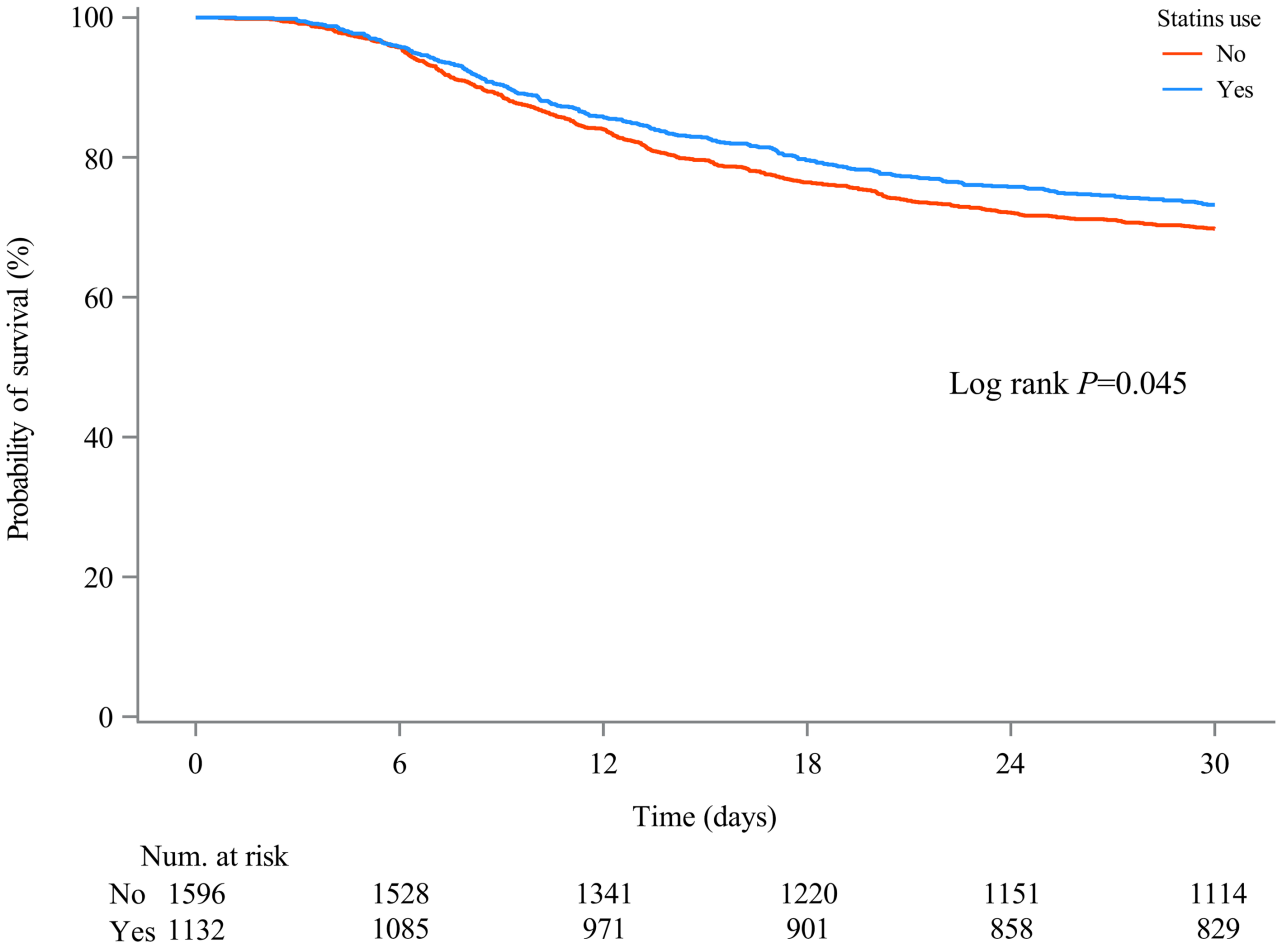
Kaplan–Meier survival curves between two groups indicated the 30-day mortality risk for the SAE patients. Non-statin users are represented by red lines, and statin users are represented by blue lines.

Table 2 shows the association between statin use and 30-day mortality in all SAE patients. Compared to SAE patients without statin treatment, statin use was associated with lower odds of 30-day mortality (HR = 0.77, 95%CI: 0.66–0.90) after adjusting for age, ethnicity, insurance, AKI, respiratory failure, cardiogenic shock, urine output, respiratory rate, temperature, CCI, SOFA, SAPS II, SIRS, WBC count, platelet count, hemoglobin level, RDW-CV, serum creatinine, anion gap, magnesium, machine ventilation, RRT, midazolam, propofol, and SAE phenotypes. After PSM, statin use was also associated with lower odds of 30-day mortality (HR = 0.83, 95%CI: 0.70–0.99) (Supplementary Table S5). The ARR was 7.15%, and the NNT was 14, indicating that 14 patients with SAE would need to be treated with statins to prevent 30-day mortality in one patient with SAE (Table 3). Compared with SAE patients without statin treatment, SAE patients treated with simvastatin treatment (HR = 0.58, 95%CI: 0.43–0.78) or atorvastatin (HR = 0.77, 95%CI: 0.65–0.93) had lower odds of 30-day mortality. Compared with SAE patients without statin treatment, 30-day mortality was lower in patients treated with statins (log-rank test *p*= 0.045) (Figure 3).

**Table 2:** Associations of statin use with 30-day mortality in all SAE patients.

Variables	Model 1		Model 2	
	HR (95%CI)	*P*	HR (95%CI)	*P*
Statins use				
No	Ref		Ref	
Yes	0.86 (0.75–0.99)	0.045	0.77 (0.66–0.90)	0.001
Type of statins				
No use	Ref		Ref	
Atorvastatin	0.90 (0.76–1.07)	0.236	0.77 (0.65–0.93)	0.006
Pravastatin	0.90 (0.58–1.37)	0.615	0.88 (0.57–1.37)	0.577
Rosuvastatin	1.50 (1.06–2.12)	0.022	1.30 (0.90–1.87)	0.156
Simvastatin	0.59 (0.44–0.79)	<0.001	0.58 (0.43–0.78)	<0.001
More than one type	0.85 (0.49–1.48)	0.569	0.77 (0.44–1.36)	0.374

Ref, Reference; HR, Hazard Ratio; CI, Confidence Interval; SAE, sepsis-associated encephalopathy. Model 1, Crude model. Model 2, Adjusted for age, ethnicity, insurance, AKI, respiratory failure, cardiogenic shock, urine output, respiratory rate, temperature, Charlson comorbidity index, SOFA, SAPS II, SIRS, WBC, platelet, hemoglobin, RDW-CV, serum creatinine, anion gap, magnesium, machine ventilation, RRT, midazolam, propofol, and SAE phenotypes.

**Table 3:** 30-day mortality in SAE patients who were treated with statins after PSM.

	Variables		30-day mortality		ARR	NNT
			Death	Survival		
Original cohort	Statin = No	1,596	482	1,114	3.37%	30
	Statin = Yes	1,133	304	829		
	Total	2,729	786	1943		
Matched cohort	Statin = No	878	289	589	5.47%	18
	Statin = Yes	878	241	637		
	Total	1756	530	1,226		
Weighted cohort	Statin = No	2759.4	896.72	1862.68	7.15%	14
	Statin = Yes	2647.64	671.14	1976.5		
	Total	5407.04	1567.86	3839.18		

SAE, sepsis-associated encephalopathy; PSM, propensity score matching; ARR, absolute risk reduction; NNT, number needed to treat.

### Associations between different statin types and 30-day mortality in patients who received statin treatment

**Figure 4:**
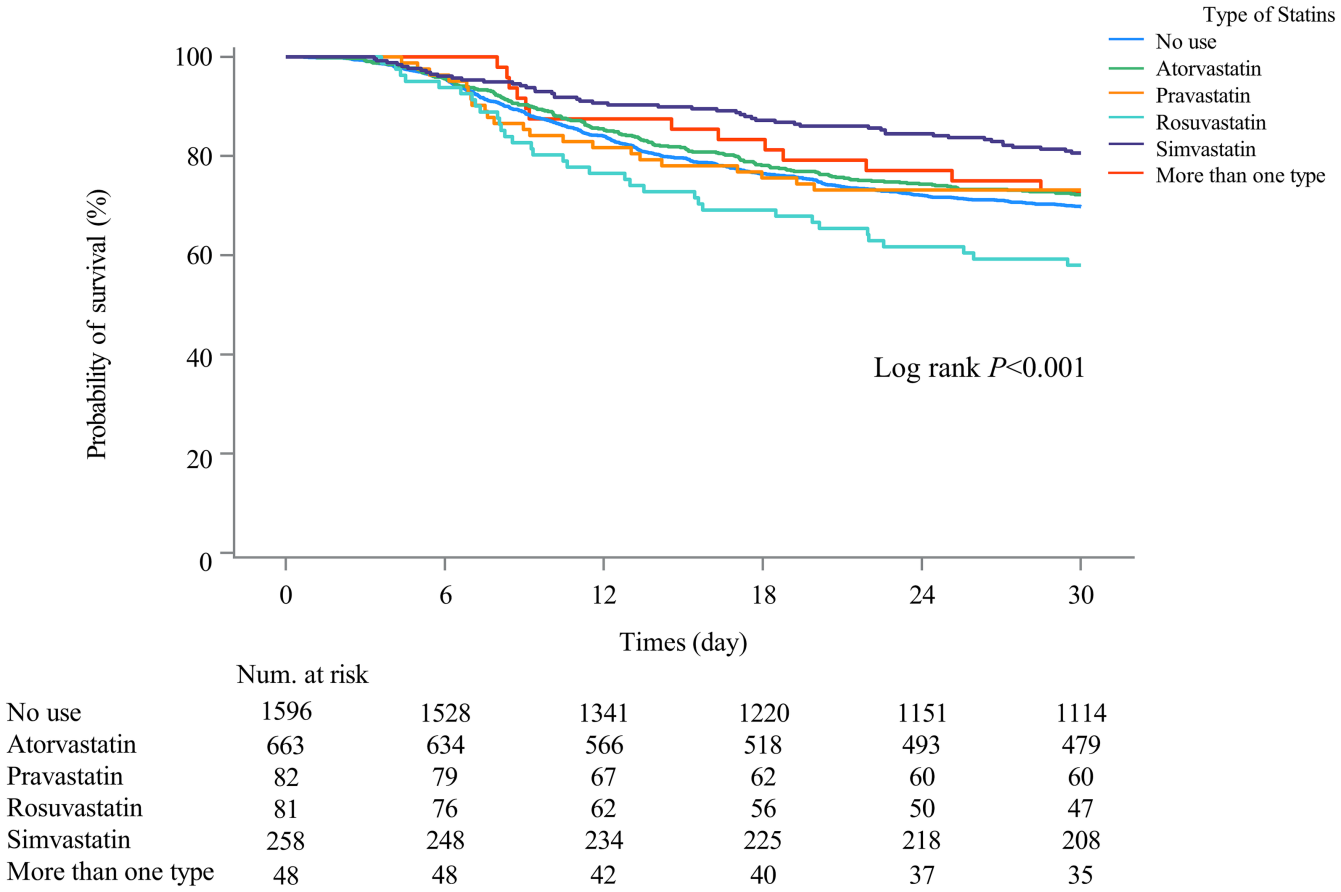
Kaplan–Meier survival curves among different types of statin user groups indicated the 30-day mortality risk for the SAE patients.

The associations of statin types with 30-day mortality in SAE patients with statin treatments were presented in Table 4. Compared with SAE patients who received atorvastatin treatment, rosuvastatin treatment was associated with higher odds of 30-day mortality (HR = 1.88, 95%CI: 1.29–2.75) in model 2. Simvastatin was of marginal significance associated with 30-day mortality (HR = 0.74, 95%CI: 0.53–1.02) in SAE patients. Additionally, no association was observed between other types of statins and 30-day mortality. Figure 4 also shows that 30-day mortality was higher in SAE patients treated with simvastatin (log-rank test *p*-value <0.001).

**Table 4:** Association of statin types with 30-day mortality in SAE patients with statin treatments.

Variables	Model 1		Model 2	
	HR (95%CI)	*P*	HR (95%CI)	*P*
Type of statins				
Atorvastatin	Ref		Ref	
Pravastatin	0.99 (0.64–1.55)	0.976	1.33 (0.84–2.09)	0.221
Rosuvastatin	1.66 (1.15–2.40)	0.006	1.88 (1.29–2.75)	0.001
Simvastatin	0.65 (0.48–0.89)	0.008	0.74 (0.53–1.02)	0.067
More than one type	0.94 (0.54–1.66)	0.843	1.01 (0.56–1.82)	0.977

Ref, reference; HR, hazard ratio; CI, confidence interval; SAE, sepsis-associated encephalopathy. Model 1: Crude model. Model 2: Adjusted for age, ethnicity, insurance, AKI, respiratory failure, cardiogenic shock, urine output, respiratory rate, temperature, Charlson comorbidity index, SOFA, SAPS II, SIRS, WBC, platelet, hemoglobin, RDW-CV, serum creatinine, anion gap, magnesium, machine ventilation, RRT, midazolam, propofol, and SAE phenotypes.

### Associations between statin use and 30-day mortality in different age, sex, SOFA, SAPS II, and SIRS groups

As shown in Table 5, compared with SAE patients without statin treatment, statin use was associated with lower odds of 30-day mortality in patients aged <65 years (HR = 0.66, 95%CI: 0.47–0.93), and SIRS <3 (HR = 0.59, 95%CI: 0.44–0.81) populations. Compared with SAE patients who received atorvastatin treatment, rosuvastatin treatment was associated with higher odds of 30-day mortality in patients with age ≥ 65 years old (HR = 1.83, 95%CI: 1.20–2.79), male (HR = 1.92, 95%CI: 1.19–3.09), SOFA ≥8 (HR = 2.06, 95%CI: 1.24–3.44), SAPS II ≥44 (HR = 2.28, 95%CI: 1.41–3.71), and SIRS ≥3 (HR = 2.22, 95%CI: 1.43–3.46) (Table 6). Furthermore, compared with SAE patients who received atorvastatin treatment, pravastatin treatment was associated with higher odds of 30-day mortality in the SOFA ≥8 (HR = 2.00, 95%CI: 1.17–3.43) and SIRS ≥3 (HR = 1.80, 95%CI: 1.03–3.13) populations.

**Table 5:** Associations of statin use with 30-day mortality in subgroups including age, sex, SOFA, SAPS II, and SIRS of all SAE patients.

Subgroups	HR (95%CI)	*P*	HR (95%CI)	*P*
Age	<65 years (*n* = 1,211)		≥65 years (*n* = 1,518)	
Statin use				
No	Ref		Ref	
Yes	0.66 (0.47–0.93)	0.017	0.83 (0.69–1.01)	0.058
Type of statins				
No use	Ref		Ref	
Atorvastatin	0.78 (0.54–1.13)	0.196	0.82 (0.66–1.01)	0.063
Pravastatin	–	–	1.12 (0.71–1.75)	0.625
Rosuvastatin	1.52 (0.65–3.54)	0.337	1.30 (0.86–1.96)	0.208
Simvastatin	0.12 (0.03–0.48)	0.003	0.72 (0.52–0.99)	0.043
More than one type	1.69 (0.39–7.30)	0.482	0.69 (0.37–1.29)	0.249
Sex	Female (*n* = 1,148)		Male (*n* = 1,581)	
Statin use				
No	Ref		Ref	
Yes	0.74 (0.58–0.95)	0.017	0.80 (0.64–0.99)	0.036
Type of statins				
No use	Ref		Ref	
Atorvastatin	0.80 (0.60–1.06)	0.124	0.78 (0.61–0.99)	0.045
Pravastatin	0.93 (0.52–1.68)	0.822	0.75 (0.38–1.48)	0.405
Rosuvastatin	1.35 (0.71–2.57)	0.365	1.26 (0.80–1.97)	0.312
Simvastatin	0.46 (0.28–0.74)	0.002	0.68 (0.46–1.01)	0.053
More than one type	0.72 (0.29–1.79)	0.482	0.82 (0.40–1.70)	0.599
SOFA	SOFA <8 (*n* = 1,258)		SOFA ≥8 (*n* = 1,471)	
Statin use				
No	Ref		Ref	
Yes	0.76 (0.59–0.98)	0.034	0.76 (0.62–0.93)	0.009
Type of statins				
No use	Ref		Ref	
Atorvastatin	0.79 (0.59–1.06)	0.122	0.76 (0.60–0.97)	0.024
Pravastatin	0.43 (0.17–1.06)	0.067	1.26 (0.76–2.10)	0.368
Rosuvastatin	1.30 (0.73–2.31)	0.371	1.32 (0.82–2.14)	0.255
Simvastatin	0.53 (0.32–0.88)	0.015	0.56 (0.38–0.82)	0.003
More than one type	1.05 (0.53–2.08)	0.884	0.47 (0.17–1.30)	0.146
SAPS II	SAPS II <44 (*n* = 1,286)		SAPS II ≥44 (*n* = 1,443)	
Statin use				
No	Ref		Ref	
Yes	0.69 (0.52–0.91)	0.008	0.80 (0.66–0.97)	0.026
Type of statins				
No use	Ref		Ref	
Atorvastatin	0.72 (0.52–0.98)	0.038	0.79 (0.63–0.99)	0.040
Pravastatin	0.86 (0.43–1.72)	0.673	0.89 (0.50–1.58)	0.698
Rosuvastatin	1.01 (0.54–1.90)	0.964	1.50 (0.95–2.36)	0.080
Simvastatin	0.43 (0.25–0.74)	0.002	0.66 (0.46–0.95)	0.026
More than one type	0.90 (0.39–2.10)	0.808	0.66 (0.31–1.42)	0.288
SIRS	SIRS <3 (*n* = 810)		SIRS ≥3 (*n* = 1919)	
Statin use				
No	Ref		Ref	
Yes	0.59 (0.44–0.81)	<0.001	0.83 (0.69–1.00)	0.051
Type of statins				
No use	Ref		Ref	
Atorvastatin	0.62 (0.44–0.88)	0.008	0.81 (0.65–1.01)	0.060
Pravastatin	0.43 (0.19–0.94)	0.035	1.26 (0.74–2.14)	0.397
Rosuvastatin	0.69 (0.32–1.47)	0.334	1.58 (1.04–2.41)	0.031
Simvastatin	0.46 (0.25–0.83)	0.010	0.64 (0.45–0.91)	0.013
More than one type	1.18 (0.50–2.77)	0.702	0.60 (0.28–1.31)	0.201

Ref, reference; HR, hazard ratio; CI, confidence interval. SOFA, sequential organ failure assessment; SAPS II, simplified acute physiology score II; SIRS, systemic inflammatory response syndrome; SAE, sepsis-associated encephalopathy. Adjusted for age (age was not adjusted in the age subgroup), ethnicity, insurance, AKI, respiratory failure, cardiogenic shock, urine output, respiratory rate, temperature, Charlson comorbidity index, SOFA (SOFA was not adjusted in the SOFA subgroup), SAPS II (SAPS II was not adjusted in SAPS II subgroup), SIRS (SIRS was not adjusted in SIRS subgroup), WBC, platelet, hemoglobin, RDW-CV, serum creatinine, anion gap, magnesium, machine ventilation, RRT, midazolam, propofol, and SAE phenotypes.

**Table 6:** Associations of statin types with 30-day mortality in subgroups including age, sex, SOFA, SAPS II, and SIRS of SAE patients with statin treatments.

Statin types	HR (95%CI)	*P*	HR (95%CI)	*P*
Age	<65 years (*n* = 1,211)		≥65 years (*n* = 1,518)	
Atorvastatin	Ref		Ref	
Pravastatin	–	–	1.55 (0.97–2.47)	0.064
Rosuvastatin	1.36 (0.49–3.73)	0.553	1.83 (1.20–2.79)	0.005
Simvastatin	0.19 (0.04–0.80)	0.023	0.86 (0.61–1.21)	0.378
More than one type	1.28 (0.11–14.63)	0.842	0.94 (0.49–1.78)	0.838
Sex	Female (*n* = 1,148)		Male (*n* = 1,581)	
Atorvastatin	Ref		Ref	
Pravastatin	1.34 (0.71–2.55)	0.368	1.12 (0.56–2.25)	0.754
Rosuvastatin	1.87 (0.93–3.76)	0.078	1.92 (1.19–3.09)	0.008
Simvastatin	0.61 (0.35–1.04)	0.067	0.76 (0.49–1.17)	0.214
More than one type	0.93 (0.36–2.42)	0.880	0.92 (0.43–1.99)	0.837
SOFA	SOFA <8 (*n* = 1,258)		SOFA ≥8 (*n* = 1,471)	
Atorvastatin	Ref		Ref	
Pravastatin	0.54 (0.21–1.36)	0.190	2.00 (1.17–3.43)	0.012
Rosuvastatin	1.75 (0.95–3.23)	0.072	2.06 (1.24–3.44)	0.006
Simvastatin	0.65 (0.38–1.14)	0.135	0.66 (0.43–1.02)	0.059
More than one type	1.19 (0.58–2.48)	0.634	0.60 (0.21–1.72)	0.345
SAPS II	SAPS II <44 (*n* = 1,286)		SAPS II ≥44 (*n* = 1,443)	
Atorvastatin	Ref		Ref	
Pravastatin	1.14 (0.55–2.33)	0.729	1.42 (0.78–2.59)	0.253
Rosuvastatin	1.40 (0.73–2.70)	0.316	2.28 (1.41–3.71)	<0.001
Simvastatin	0.54 (0.30–0.97)	0.038	0.84 (0.56–1.26)	0.407
More than one type	1.13 (0.46–2.77)	0.790	0.90 (0.40–2.02)	0.793
SIRS	SIRS <3 (*n* = 810)		SIRS ≥3 (*n* = 1919)	
Atorvastatin	Ref		Ref	
Pravastatin	0.81 (0.35–1.87)	0.625	1.80 (1.03–3.13)	0.039
Rosuvastatin	1.37 (0.59–3.18)	0.464	2.22 (1.43–3.46)	<0.001
Simvastatin	0.65 (0.34–1.27)	0.208	0.84 (0.57–1.24)	0.388
More than one type	1.51 (0.59–3.88)	0.393	0.83 (0.37–1.87)	0.655

Ref, reference; HR, hazard ratio; CI, confidence interval; SOFA, sequential organ failure assessment; SAPS II, simplified acute physiology score II; SIRS, systemic inflammatory response syndrome; SAE, sepsis-associated encephalopathy. Adjusted for age (age was not adjusted in the age subgroup), ethnicity, insurance, AKI, respiratory failure, cardiogenic shock, urine output, respiratory rate, temperature, Charlson comorbidity index, SOFA (SOFA was not adjusted in the SOFA subgroup), SAPS II (SAPS II was not adjusted in the SAP SII subgroup), SIRS (SIRS was not adjusted in the SIRS subgroup), WBC, platelet, hemoglobin, RDW-CV, serum creatinine, anion gap, magnesium, machine ventilation, RRT, midazolam, propofol, and SAE phenotypes.

## Discussion

We investigated the association between statin use and 30-day mortality among patients with SAE. Our findings indicated that statin use was associated with a lower 30-day mortality risk in all SAE patients. Furthermore, when compared to patients who received atorvastatin treatment, rosuvastatin treatment was associated with higher odds of 30-day mortality in SAE patients who received statin treatment. An association between statin use and a lower risk of 30-day mortality was also found in SAE patients aged <65 years old and SIRS <3. Furthermore, the findings revealed a relationship between rosuvastatin treatment and a higher risk of 30-day mortality in specific patient groups, including those aged ≥65 years old, male, SOFA ≥8, SAPS II ≥44, and SIRS ≥3.

In the SAE population, our findings revealed a potential benefit associated with statin use. The findings were consistent with some existing studies that suggested that statins, through their pleiotropic effects beyond lipid-lowering, may have potential immunomodulatory and anti-inflammatory properties that could be beneficial in sepsis (20–22). In the case of sepsis patients admitted to the medical ICU, the usage of statins before ICU admission was causally linked to a reduction in both 30-day ICU mortality and 30-day in-hospital mortality, compared to non-use (22). Pienkos et al. also reported that in sepsis-related acute respiratory distress syndrome, simvastatin therapy seems safe and could reduce mortality (23). However, it is crucial to note that the studies on the overall impact of statins in sepsis are mixed, with some studies reporting no significant survival benefit (24). Azkarate et al. reported that the administration of statin therapy before hospitalization did not significantly influence the manifestation of sepsis or its outcomes (24). A meta-analysis also found that statin therapy for the indication of sepsis is not recommended (25). Further research is required to confirm the role of statins in SAE patients. Careful consideration should be given when choosing a specific statin.

Rosuvastatin was associated with a higher 30-day mortality rate in certain subgroups, including older patients (aged 65 years and older), males, and those with higher SOFA scores ≥8, SAPS II scores ≥44, and SIRS scores ≥3. The finding emphasizes the need for more nuanced, individualized treatment strategies in critically ill patients, particularly in those with higher comorbidity and severity of disease. In older patients, age-related changes in pharmacokinetics and pharmacodynamics, as well as altered immune responses, may amplify the adverse effects of statins, including their potential to increase systemic inflammation or interfere with mitochondrial function in neuronal tissue, which could worsen outcomes in SAE. In males, hormonal differences could influence statin metabolism and its impact on immune modulation. The interplay between the sex-specific immune response and statin therapy may further contribute to adverse outcomes, but further targeted research is required to elucidate these mechanisms fully. For patients with higher SOFA and SAPS II scores, which indicate greater organ dysfunction and physiological derangement, the potential for statin-induced immunosuppression or mitochondrial toxicity could be more pronounced, further compounding the already heightened risk of mortality. This suggests that in patients with severe sepsis or septic shock, rosuvastatin may exacerbate organ dysfunction and inflammatory imbalance. To mitigate these risks, clinicians may consider tailoring statin therapy based on patient age, sex, and severity of illness, carefully considering the potential benefits of statin use in SAE against the risks of worsening outcomes in vulnerable subgroups. Alternative therapeutic strategies or dose adjustments may be warranted for high-risk patients, alongside close monitoring for adverse effects.

The choices of statin in clinical practice are influenced by various factors, including drug cost, the patient’s comorbid conditions, and the severity of the disease. Cost considerations are particularly relevant in resource-limited settings where the price disparity between atorvastatin and rosuvastatin could impact treatment choices (26). Additionally, patients with multiple underlying conditions may require a statin with a more favorable side effect profile, potentially influencing the choice of rosuvastatin despite its higher cost (27). In the context of SAE, where patients often present with severe systemic inflammation and multi-organ dysfunction, the choice of statin may need to account for its anti-inflammatory effects and ability to penetrate the blood–brain barrier. The differential outcomes associated with lipophilic (simvastatin) and hydrophilic (rosuvastatin) statins may be explained by their ability to penetrate the blood–brain barrier and modulate neuroinflammation, which are critical factors in SAE pathophysiology. Atorvastatin’s tissue penetration may offer additional benefits in such severe conditions compared to rosuvastatin, which could explain the observed differences in mortality (28).

Compared with atorvastatin use in SAE patients, the association of pravastatin or simvastatin with 30-day mortality was not statistically different. It is noteworthy that the association between rosuvastatin and higher odds of 30-day mortality was observed in statin users. This finding indicated caution against the use of rosuvastatin. Lee et al. (11) also demonstrated that compared with atorvastatin and simvastatin, rosuvastatin had an inferior effectiveness in reducing mortality. Pienkos et al. (23) also found that rosuvastatin was associated with an increased mortality in low-cholesterol patients. The observed differences may be attributed to variations in the population studied and differences in statin dosages. Furthermore, differences in pharmacokinetic and pharmacodynamic properties between statins contribute to variations in outcomes. The hydrophilic nature of rosuvastatin may influence its distribution and tissue penetration, compared to the more lipophilic atorvastatin, potentially impacting their respective efficacy in SAE (29). The underlying reasons for the potential differential effects of statins require further investigation in SAE, particularly considering the complexity of the underlying pathophysiology.

The pathophysiological factors of SAE included mainly neuroinflammation, impairment of the blood–brain barrier, disorders of brain perfusion, changes in neurotransmitters, and alterations in neuroanatomy (3). Statins may exert beneficial effects through their pleiotropic properties, including anti-inflammatory and immune-modulatory effects (30, 31). Statin has been shown to suppress pro-inflammatory cytokines such as interleukin-1 (IL-1), IL-6, and tumor necrosis factor-*α*, and these three cytokines are theorized to play important roles in SAE initiation (32, 33). Furthermore, statins have been shown to exert neuroprotective effects through their ability to modulate neuronal apoptosis, reduce oxidative stress, and improve cerebral blood flow (34).

The finding that statin treatments were associated with lower 30-day mortality compared in SAE patients without statin treatments has implications for clinical practice. Therefore, using statins with caution needs to be considered in the assessment and management of SAE patients to improve patients’ prognosis. Rosuvastatin should be avoided in SAP patients of those aged ≥65 years, males, SOFA ≥8, SAPS II ≥44, and SIRS ≥3. Furthermore, it is imperative to conduct randomized trials or prospective studies and delve into the underlying mechanisms to confirm and provide a better understanding of these findings.

The study is subject to certain limitations. First, this study was conducted solely at a single medical facility, which may present limitations in terms of generalizability. Future investigations encompassing multiple centers and larger patient cohorts should be undertaken to validate the results. Second, our study did not assess all types of statin use, as only atorvastatin, pravastatin, rosuvastatin, and simvastatin were used. The lack of dosage and duration data limits actionable clinical insights. It remains unclear whether high doses or longer courses of simvastatin or atorvastatin amplify benefits, or if dose adjustments could mitigate rosuvastatin-associated risks. The absence of comprehensive blood lipid levels data before and after treatment limits further analysis. Finally, the covariates utilized for analysis were solely those available within the database, and there may exist certain unmeasured confounders. Therefore, we emphasize the importance of future studies incorporating an expanded set of covariates to enhance the comprehensiveness of the analysis and strengthen the overall scientific validity of the study.

## Conclusion

Our study found an association between statin use and a lower risk of 30-day mortality in SAE patients. Caution should be exercised in the choice of statin, as rosuvastatin was associated with higher odds of 30-day mortality compared to SAE patients with atorvastatin treatment. Furthermore, it is imperative to conduct prospective studies and delve into the underlying mechanisms to confirm and provide a better understanding of these findings.

## Data Availability

The data analyzed in this study was obtained from the Medical Information Mart for Intensive Care IV (MIMIC-IV), the following licenses/restrictions apply: To access the files, users must be credentialed users, complete the required training (CITI Data or Specimens Only Research) and sign the data use agreement for the project. Requests to access these datasets should be directed to PhysioNet, https://physionet.org/, DOI: 10.13026/6 mm1-ek67.
