# Supplementary material for: Association between statin use and 30-day mortality among patients with sepsis-associated encephalopathy: a retrospective cohort study
Source: Front Neurol. 2024 Dec 27;15:1371314. doi: 10.3389/fneur.2024.1371314 (PMC11720963; doi:10.3389/fneur.2024.1371314)
Supplement: Supplementary file 1 [file Supplementary_file_1.docx]

**Supplementary Table 1.** The standardized of statins dose

| Variables | Model 1 | | Model 2 | |  |
| --- | --- | --- | --- | --- | --- |
|  | HR (95% CI) | *P* | HR (95% CI) | *P* | N(%) |
| dose |  |  |  |  |  |
| No use | Ref |  | Ref |  | 1596(59.53) |
| Atorvastatin High dose | 0.95 (0.79-1.14) | 0.553 | 0.83 (0.68-1.01) | 0.057 | 518(19.32) |
| Atorvastatin Low dose | 0.77 (0.55-1.09) | 0.139 | 0.63 (0.44-0.90) | 0.01 | 146(5.45) |
| Pravastatin High dose | 1.10 (0.54-2.20) | 0.798 | 0.97 (0.48-1.97) | 0.931 | 25(0.93) |
| Pravastatin Low dose | 0.81 (0.48-1.38) | 0.441 | 0.84 (0.49-1.45) | 0.531 | 57(2.13) |
| Rosuvastatin High dose | 1.41 (0.97-2.06) | 0.07 | 1.23 (0.83-1.81) | 0.302 | 72(2.69) |
| Rosuvastatin Low dose | 2.30 (0.95-5.55) | 0.064 | 1.96 (0.80-4.80) | 0.142 | 9(0.34) |
| Simvastatin High dose | 0.60 (0.33-1.10) | 0.099 | 0.59 (0.32-1.09) | 0.09 | 55(2.05) |
| Simvastatin Low dose | 0.59 (0.42-0.81) | 0.001 | 0.57 (0.41-0.80) | 0.001 | 203(7.57) |

Ref: Reference, HR: Hazard Ratio, CI: Confidence Interval.

SAE: sepsis-associated encephalopathy.

Model 1: Crude model.

Model 2: Adjusted for age, ethnicity, insurance, AKI, respiratory failure, cardiogenic shock, urine output, respiratory rate, temperature, Charlson comorbidity index, SOFA, SAPS II, SIRS, WBC, platelet, hemoglobin, RDW-CV, serum creatinine, anion gap, magnesium, machine ventilation, RRT, midazolam, propofol, and SAE phenotypes.

**Supplementary Table 2.** The distribution of missing values

| Variables | n (%) |
| --- | --- |
| AKI | 1 (0.04) |
| Urine output | 102 (3.74) |
| Respiratory rate | 107 (3.92) |
| Temperature | 95 (3.48) |
| WBC | 20 (0.73) |
| Platelet | 20 (0.73) |
| Hemoglobin | 19 (0.70) |
| RDW | 20 (0.73) |
| Serum creatinine | 12 (0.44) |
| Anion gap | 14 (0.51) |
| Magnesium | 44 (1.61) |

AKI: Acute kidney injury; WBC: White blood cell; RDW: Red blood cell distribution width.

**Supplementary Table 3.** Sensitivity analysis before and after interpolation of missing values

| Variables | Before interpolation | After interpolation | Statistics | P |
| --- | --- | --- | --- | --- |
| AKI, n (%) |  |  | χ^2^=0.001 | 0.992 |
| No | 911 (33.39) | 911 (33.38) |  |  |
| Yes | 1817 (66.61) | 1818 (66.62) |  |  |
| Urine output, ml, M (Q1, Q3) | 1310.00 (769.00, 2090.00) | 1300.00 (750.00, 2085.00) | Z=0.693 | 0.488 |
| Respiratory rate, insp/min, Mean ± SD | 21.10 ± 6.83 | 21.07 ± 6.85 | t=0.18 | 0.861 |
| Temperature, Deg.C, Mean ± SD | 36.76 ± 0.96 | 36.76 ± 0.96 | t=0.06 | 0.951 |
| WBC, k/μL, M (Q1, Q3) | 12.00 (8.30, 17.00) | 12.00 (8.30, 17.00) | Z=0.004 | 0.997 |
| Platelet, k/μL, M (Q1, Q3) | 185.00 (124.00, 258.00) | 185.00 (124.00, 258.00) | Z=-0.008 | 0.993 |
| Hemoglobin, g/dL, Mean ± SD | 10.45 ± 2.37 | 10.45 ± 2.37 | t=-0.07 | 0.944 |
| RDW, %, Mean ± SD | 15.73 ± 2.66 | 15.73 ± 2.66 | t=0.01 | 0.992 |
| Serum Creatinine, mg/dL, M (Q1, Q3) | 1.20 (0.80, 2.00) | 1.20 (0.80, 2.00) | Z=-0.038 | 0.970 |
| Anion gap, meq/L, Mean ± SD | 15.85 ± 4.89 | 15.85 ± 4.89 | t=-0.04 | 0.967 |
| Magnesium, mg/dL, Mean ± SD | 2.03 ± 0.50 | 2.03 ± 0.49 | t=0.24 | 0.813 |

SD: Standard Deviation; M: Median; Q₁: 1st Quartile; Q₃: 3st Quartile; t: Student's t test; W: Wilcoxon rank sum test; χ^2^: Chi-square test.

AKI: Acute kidney injury; WBC: White blood cell; RDW: Red blood cell distribution width.

Supplementary Table 4. The basic characteristic of patients with SAE after PSM

|  | Original cohort | | | | Matched cohort | | | | Weighted cohort | | | |
| --- | --- | --- | --- | --- | --- | --- | --- | --- | --- | --- | --- | --- |
| Variables | Statin=No (n=1596) | Statin=Yes (n=1133) | *P* | SMD | Statin=No (n=878) | Statin=Yes (n=878) | *P* | SMD | Statin=No (n=2759.40) | Statin=Yes (n=2647.64) | *P* | SMD |
| Age, years, Mean (±SD) | 60.94 (±16.36) | 70.35 (±11.23) | <0.001 | 0.671 | 68.69 (±12.79) | 68.56 (±11.35) | 0.834 | 0.010 | 65.21 (±15.79) | 66.38 (±12.74) | 0.079 | 0.081 |
| Gender, n (%) |  |  | 0.097 | 0.066 |  |  | 0.439 | 0.039 |  |  | 0.089 | 0.075 |
| Female | 693 (43.42) | 455 (40.16) |  |  | 379 (43.17) | 362 (41.23) |  |  | 1195.25 (43.32) | 1049.71 (39.65) |  |  |
| Male | 903 (56.58) | 678 (59.84) |  |  | 499 (56.83) | 516 (58.77) |  |  | 1564.15 (56.68) | 1597.93 (60.35) |  |  |
| Ethnicity, n (%) |  |  | 0.044 | 0.111 |  |  | 0.466 | 0.076 |  |  | 0.933 | 0.029 |
| White | 1024 (64.16) | 763 (67.34) |  |  | 603 (68.68) | 582 (66.29) |  |  | 1799.45 (65.21) | 1755.93 (66.32) |  |  |
| Black | 139 (8.71) | 114 (10.06) |  |  | 77 (8.77) | 96 (10.93) |  |  | 264.07 (9.57) | 256.94 (9.70) |  |  |
| Other | 188 (11.78) | 105 (9.27) |  |  | 84 (9.57) | 82 (9.34) |  |  | 296.01 (10.73) | 272.56 (10.29) |  |  |
| Unknown | 245 (15.35) | 151 (13.33) |  |  | 114 (12.98) | 118 (13.44) |  |  | 399.88 (14.49) | 362.22 (13.68) |  |  |
| Insurance, n (%) |  |  | <0.001 | 0.375 |  |  | 0.613 | 0.047 |  |  | <0.001 | 0.182 |
| Medicaid | 164 (10.28) | 33 (2.91) |  |  | 26 (2.96) | 33 (3.76) |  |  | 223.94 (8.12) | 101.98 (3.85) |  |  |
| Medicare | 678 (42.48) | 645 (56.93) |  |  | 484 (55.13) | 473 (53.87) |  |  | 1345.56 (48.76) | 1375.73 (51.96) |  |  |
| Other | 754 (47.24) | 455 (40.16) |  |  | 368 (41.91) | 372 (42.37) |  |  | 1189.90 (43.12) | 1169.93 (44.19) |  |  |
| Marital status, n (%) |  |  | <0.001 | 0.164 |  |  | 0.933 | 0.018 |  |  | 0.046 | 0.108 |
| Married | 655 (41.04) | 536 (47.31) |  |  | 407 (46.36) | 402 (45.79) |  |  | 1187.32 (43.03) | 1196.26 (45.18) |  |  |
| Unknown | 196 (12.28) | 92 (8.12) |  |  | 73 (8.31) | 77 (8.77) |  |  | 312.11 (11.31) | 215.57 (8.14) |  |  |
| Unmarried | 745 (46.68) | 505 (44.57) |  |  | 398 (45.33) | 399 (45.44) |  |  | 1259.97 (45.66) | 1235.81 (46.68) |  |  |
| AKI, n (%) |  |  | 0.580 | 0.023 |  |  | 1.000 | <0.001 |  |  | 0.075 | 0.077 |
| No | 540 (33.83) | 371 (32.74) |  |  | 288 (32.8) | 288 (32.8) |  |  | 949.44 (34.41) | 816.10 (30.82) |  |  |
| Yes | 1056 (66.17) | 762 (67.26) |  |  | 590 (67.2) | 590 (67.2) |  |  | 1809.96 (65.59) | 1831.54 (69.18) |  |  |
| Respiratory failure, n (%) |  |  | 0.313 | 0.041 |  |  | 0.808 | 0.014 |  |  | 0.878 | 0.007 |
| No | 633 (39.66) | 472 (41.66) |  |  | 361 (41.12) | 355 (40.43) |  |  | 1108.12 (40.16) | 1071.90 (40.49) |  |  |
| Yes | 963 (60.34) | 661 (58.34) |  |  | 517 (58.88) | 523 (59.57) |  |  | 1651.28 (59.84) | 1575.74 (59.51) |  |  |
| Cardiogenic shock, n (%) |  |  | <0.001 | 0.175 |  |  | 1.000 | <0.001 |  |  | 0.920 | 0.005 |
| No | 1559 (97.68) | 1068 (94.26) |  |  | 846 (96.36) | 846 (96.36) |  |  | 2653.91 (96.18) | 2548.71 (96.26) |  |  |
| Yes | 37 (2.32) | 65 (5.74) |  |  | 32 (3.64) | 32 (3.64) |  |  | 105.50 (3.82) | 98.93 (3.74) |  |  |
| Urine output, mL, M (Q_1_, Q_3_) | 1300 (729.25-2076.25) | 1300 (757-2090) | 0.628 | 0.028 | 1262 (760.75-1955) | 1290 (738.25-2090) | 0.473 | 0.025 | 1294.76 (738.19-2038.80) | 1272.05 (725.49-2066.97) | 0.842 | 0.029 |
| Heart rate, bpm, Mean (±SD) | 97.45 (±22.74) | 89.13 (±20.39) | <0.001 | 0.385 | 91.52 (±20.87) | 90.73 (±21.04) | 0.432 | 0.038 | 95.89 (±22.56) | 90.44 (±20.60) | <0.001 | 0.252 |
| Respiratory rate, insp/min, Mean (±SD) | 21.25 (±7.04) | 20.81 (±6.57) | 0.095 | 0.065 | 20.99 (±6.91) | 20.96 (±6.55) | 0.941 | 0.004 | 21.13 (±6.90) | 20.79 (±6.59) | 0.236 | 0.050 |
| Temperature, Deg.C, Mean (±SD) | 36.76 (±0.99) | 36.77 (±0.91) | 0.779 | 0.011 | 36.76 (±0.91) | 36.78 (±0.91) | 0.736 | 0.016 | 36.76 (±0.96) | 36.76 (±0.97) | 0.920 | 0.005 |
| GCS, Mean (±SD) | 11.10 (±3.81) | 11.40 (±3.87) | 0.049 | 0.076 | 11.38 (±3.55) | 11.35 (±3.90) | 0.873 | 0.008 | 11.34 (±3.62) | 10.92 (±4.19) | 0.024 | 0.108 |
| Charlson comorbidity index, Mean (±SD) | 3.17 (±2.44) | 4.04 (±2.39) | <0.001 | 0.359 | 3.70 (±2.54) | 3.82 (±2.33) | 0.313 | 0.048 | 3.58 (±2.64) | 3.60 (±2.32) | 0.838 | 0.009 |
| SOFA, Mean (±SD) | 8.89 (±4.29) | 7.98 (±3.69) | <0.001 | 0.230 | 7.95 (±3.91) | 8.03 (±3.73) | 0.671 | 0.020 | 8.46 (±4.10) | 8.38 (±3.87) | 0.648 | 0.021 |
| SAPS II, Mean (±SD) | 46.62 (±16.53) | 46.51 (±15.06) | 0.866 | 0.007 | 46.43 (±15.40) | 46.52 (±15.35) | 0.900 | 0.006 | 46.78 (±15.73) | 47.39 (±16.50) | 0.440 | 0.037 |
| SIRS, Mean (±SD) | 2.97 (±0.86) | 2.81 (±0.90) | <0.001 | 0.183 | 2.86 (±0.85) | 2.84 (±0.89) | 0.702 | 0.018 | 2.90 (±0.87) | 2.88 (±0.88) | 0.596 | 0.023 |
| WBC, k/μL, M (Q_1_, Q_3_) | 11.9 (7.88-17.12) | 12.3 (8.7-16.8) | 0.126 | 0.014 | 12 (8.1-16.8) | 12.3 (8.7-16.88) | 0.621 | 0.047 | 11.80 (7.90-16.80) | 12.50 (8.90-17.70) | 0.002 | 0.052 |
| Platelet, k/μL, Mean (±SD) | 195.91 (±123.11) | 213.80 (±112.24) | <0.001 | 0.152 | 217.00 (±126.75) | 213.83 (±104.32) | 0.567 | 0.027 | 200.19 (±121.12) | 218.02 (±116.27) | 0.001 | 0.150 |
| Hemoglobin, g/dL, Mean (±SD) | 10.52 (±2.42) | 10.36 (±2.31) | 0.070 | 0.071 | 10.49 (±2.23) | 10.50 (±2.34) | 0.879 | 0.007 | 10.47 (±2.35) | 10.51 (±2.41) | 0.719 | 0.016 |
| RDW-CV, %, Mean (±SD) | 15.91 (±2.87) | 15.47 (±2.31) | <0.001 | 0.169 | 15.56 (±2.36) | 15.55 (±2.40) | 0.936 | 0.004 | 15.72 (±2.65) | 15.70 (±2.65) | 0.875 | 0.008 |
| Serum Creatinine, mg/dL, M (Q_1_, Q_3_) | 1.1 (0.8-2) | 1.2 (0.9-2) | <0.001 | 0.038 | 1.1 (0.8-1.9) | 1.2 (0.83-2.1) | 0.001 | 0.068 | 1.20 (0.80-2.00) | 1.20 (0.80-2.00) | 0.009 | 0.012 |
| Anion gap, meq/L, Mean (±SD) | 16.11 (±5.02) | 15.49 (±4.66) | 0.001 | 0.129 | 15.61 (±4.68) | 15.68 (±4.79) | 0.751 | 0.015 | 16.05 (±4.96) | 15.78 (±5.23) | 0.364 | 0.053 |
| Magnesium, mg/dL, M (Q_1_, Q_3_) | 1.9 (1.7-2.2) | 2 (1.8-2.3) | <0.001 | 0.243 | 2 (1.8-2.2) | 2 (1.8-2.3) | 0.220 | 0.045 | 2.00 (1.70-2.20) | 2.00 (1.80-2.30) | <0.001 | 0.177 |
| Machine ventilation, n (%) |  |  | 0.001 | 0.134 |  |  | 0.390 | 0.044 |  |  | 0.010 | 0.120 |
| No | 338 (21.18) | 181 (15.98) |  |  | 170 (19.36) | 155 (17.65) |  |  | 580.28 (21.03) | 432.79 (16.35) |  |  |
| Yes | 1258 (78.82) | 952 (84.02) |  |  | 708 (80.64) | 723 (82.35) |  |  | 2179.13 (78.97) | 2214.85 (83.65) |  |  |
| Vasopressors, n (%) |  |  | 0.045 | 0.079 |  |  | 0.962 | 0.005 |  |  | 0.001 | 0.152 |
| No | 883 (55.33) | 582 (51.37) |  |  | 471 (53.64) | 469 (53.42) |  |  | 1558.86 (56.49) | 1295.54 (48.93) |  |  |
| Yes | 713 (44.67) | 551 (48.63) |  |  | 407 (46.36) | 409 (46.58) |  |  | 1200.54 (43.51) | 1352.10 (51.07) |  |  |
| RRT, n (%) |  |  | 0.301 | 0.043 |  |  | 0.496 | 0.037 |  |  | 0.522 | 0.032 |
| No | 1450 (90.85) | 1043 (92.06) |  |  | 807 (91.91) | 798 (90.89) |  |  | 2520.80 (91.35) | 2394.67 (90.45) |  |  |
| Yes | 146 (9.15) | 90 (7.94) |  |  | 71 (8.09) | 80 (9.11) |  |  | 238.61 (8.65) | 252.97 (9.55) |  |  |
| Midazolam, n (%) |  |  | <0.001 | 0.157 |  |  | 1.000 | 0.003 |  |  | 0.618 | 0.023 |
| No | 1180 (73.93) | 912 (80.49) |  |  | 695 (79.16) | 694 (79.04) |  |  | 2117.21 (76.73) | 2005.27 (75.74) |  |  |
| Yes | 416 (26.07) | 221 (19.51) |  |  | 183 (20.84) | 184 (20.96) |  |  | 642.19 (23.27) | 642.37 (24.26) |  |  |
| Propofol, n (%) |  |  | 0.499 | 0.028 |  |  | 0.961 | 0.005 |  |  | 0.847 | 0.008 |
| No | 888 (55.64) | 646 (57.02) |  |  | 524 (59.68) | 522 (59.45) |  |  | 1562.30 (56.62) | 1510.00 (57.03) |  |  |
| Yes | 708 (44.36) | 487 (42.98) |  |  | 354 (40.32) | 356 (40.55) |  |  | 1197.10 (43.38) | 1137.64 (42.97) |  |  |
| Dexmedetomidine, n (%) |  |  | 0.955 | 0.005 |  |  | 0.835 | 0.015 |  |  | 0.854 | 0.008 |
| No | 1501 (94.05) | 1067 (94.17) |  |  | 831 (94.65) | 828 (94.31) |  |  | 2600.46 (94.24) | 2490.09 (94.05) |  |  |
| Yes | 95 (5.95) | 66 (5.83) |  |  | 47 (5.35) | 50 (5.69) |  |  | 158.94 (5.76) | 157.55 (5.95) |  |  |
| Type of statins, n (%) |  |  | <0.001 | 1.189 |  |  | <0.001 | 1.154 |  |  | <0.001 | 1.152 |
| Atorvastatin | 0 (0) | 664 (58.61) |  |  | 0 (0) | 527 (60.02) |  |  | 0.00 (0.00) | 1591.43 (60.11) |  |  |
| More than one type | 0 (0) | 48 (4.24) |  |  | 0 (0) | 29 (3.3) |  |  | 0.00 (0.00) | 91.72 (3.46) |  |  |
| No use | 1596 (100) | 0 (0) |  |  | 878 (100) | 0 (0) |  |  | 2759.40 (100.00) | 0.00 (0.00) |  |  |
| Pravastatin | 0 (0) | 82 (7.24) |  |  | 0 (0) | 55 (6.26) |  |  | 0.00 (0.00) | 178.64 (6.75) |  |  |
| Rosuvastatin | 0 (0) | 81 (7.15) |  |  | 0 (0) | 59 (6.72) |  |  | 0.00 (0.00) | 176.20 (6.65) |  |  |
| Simvastatin | 0 (0) | 258 (22.77) |  |  | 0 (0) | 208 (23.69) |  |  | 0.00 (0.00) | 609.65 (23.03) |  |  |
| SAE phenotypes, n (%) |  |  | 0.025 | 0.119 |  |  | 0.914 | 0.034 |  |  | 0.005 | 0.164 |
| Ischemic-hypoxic SAE | 323 (20.24) | 282 (24.89) |  |  | 188 (21.41) | 199 (22.67) |  |  | 526.44 (19.08) | 676.13 (25.54) |  |  |
| Metabolic SAE | 269 (16.85) | 180 (15.89) |  |  | 142 (16.17) | 142 (16.17) |  |  | 482.37 (17.48) | 392.84 (14.84) |  |  |
| Mixed SAE | 161 (10.09) | 95 (8.38) |  |  | 77 (8.77) | 79 (9) |  |  | 270.44 (9.80) | 269.78 (10.19) |  |  |
| Unclassified SAE | 843 (52.82) | 576 (50.84) |  |  | 471 (53.64) | 458 (52.16) |  |  | 1480.16 (53.64) | 1308.89 (49.44) |  |  |
| Survival time, day, M (Q_1_, Q_3_) | 30 (20.05-30) | 30 (25.35-30) | 0.046 | 0.024 | 30 (18.9-30) | 30 (24.61-30) | 0.018 | 0.027 | 30.00 (17.46-30.00) | 30.00 (29.23-30.00) | <0.001 | 0.012 |
| Days30 mortality, n (%) |  |  | 0.061 | 0.075 |  |  | 0.015 | 0.119 |  |  | <0.001 | 0.158 |
| Survival | 1114 (69.8) | 829 (73.17) |  |  | 589 (67.08) | 637 (72.55) |  |  | 1862.68 (67.50) | 1976.50 (74.65) |  |  |
| Death | 482 (30.2) | 304 (26.83) |  |  | 289 (32.92) | 241 (27.45) |  |  | 896.72 (32.50) | 671.14 (25.35) |  |  |
| dose, n (%) |  |  | <0.001 | 2.296 |  |  | <0.001 | 2.392 |  |  | <0.001 | 2.280 |
| No use | 1596 (100) | 0 (0) |  |  | 878 (100) | 0 (0) |  |  | 2759.40 (100.00) | 0.00 (0.00) |  |  |
| Atorvastatin High dose | 0 (0) | 518 (45.72) |  |  | 0 (0) | 414 (47.15) |  |  | 0.00 (0.00) | 1242.67 (48.62) |  |  |
| Atorvastatin Low dose | 0 (0) | 146 (12.89) |  |  | 0 (0) | 113 (12.87) |  |  | 0.00 (0.00) | 348.76 (13.65) |  |  |
| Pravastatin High dose | 0 (0) | 25 (2.21) |  |  | 0 (0) | 18 (2.05) |  |  | 0.00 (0.00) | 58.21 (2.28) |  |  |
| Pravastatin Low dose | 0 (0) | 57 (5.03) |  |  | 0 (0) | 37 (4.21) |  |  | 0.00 (0.00) | 120.43 (4.71) |  |  |
| Rosuvastatin High dose | 0 (0) | 72 (6.35) |  |  | 0 (0) | 52 (5.92) |  |  | 0.00 (0.00) | 153.62 (6.01) |  |  |
| Rosuvastatin Low dose | 0 (0) | 9 (0.79) |  |  | 0 (0) | 7 (0.8) |  |  | 0.00 (0.00) | 22.57 (0.88) |  |  |
| Simvastatin High dose | 0 (0) | 55 (4.85) |  |  | 0 (0) | 34 (3.87) |  |  | 0.00 (0.00) | 123.35 (4.83) |  |  |
| Simvastatin Low dose | 0 (0) | 203 (17.92) |  |  | 0 (0) | 174 (19.82) |  |  | 0.00 (0.00) | 486.30 (19.03) |  |  |

Supplementary Table 5. Associations of statins use with 30-day mortality in all SAE patients after PSM

| Variables | Original cohort | | | | Matched cohort | | | | Weighted cohort | |
| --- | --- | --- | --- | --- | --- | --- | --- | --- | --- | --- |
|  | Model 1 | | Model 2 | | Model 1 | | Model 2 | | Model 2 | |
|  | HR (95% CI) | *P* | HR (95% CI) | *P* | HR (95% CI) | *P* | HR (95% CI) | *P* | HR (95% CI) | *P* |
| Statins use |  |  |  |  |  |  |  |  |  |  |
| No | Ref |  | Ref |  | Ref |  | Ref |  | Ref |  |
| Yes | 0.87 (0.75-1.00) | 0.050 | 0.76 (0.65-0.89) | 0.001 | 0.81 (0.68-0.96) | 0.015 | 0.83 (0.70-0.99) | 0.037 | 0.77 (0.65-0.91) | 0.003 |

HR: hazard ratio; CI: confidence intervals; Ref: reference.

Model 1 was crude model;

Original cohort model 2 adjusting age, race, respiratory failure, cardiogenic shock, urine output, temperature, charlson comorbidity index, SOFA, SAPSII, sirs, hemoglobin, RDW, creatinine blood, midazolam, propofol;

Matched cohort model 2 adjusting age, insurance, AKI, respiratory failure, cardiogenic shock, urine output, and temperature.


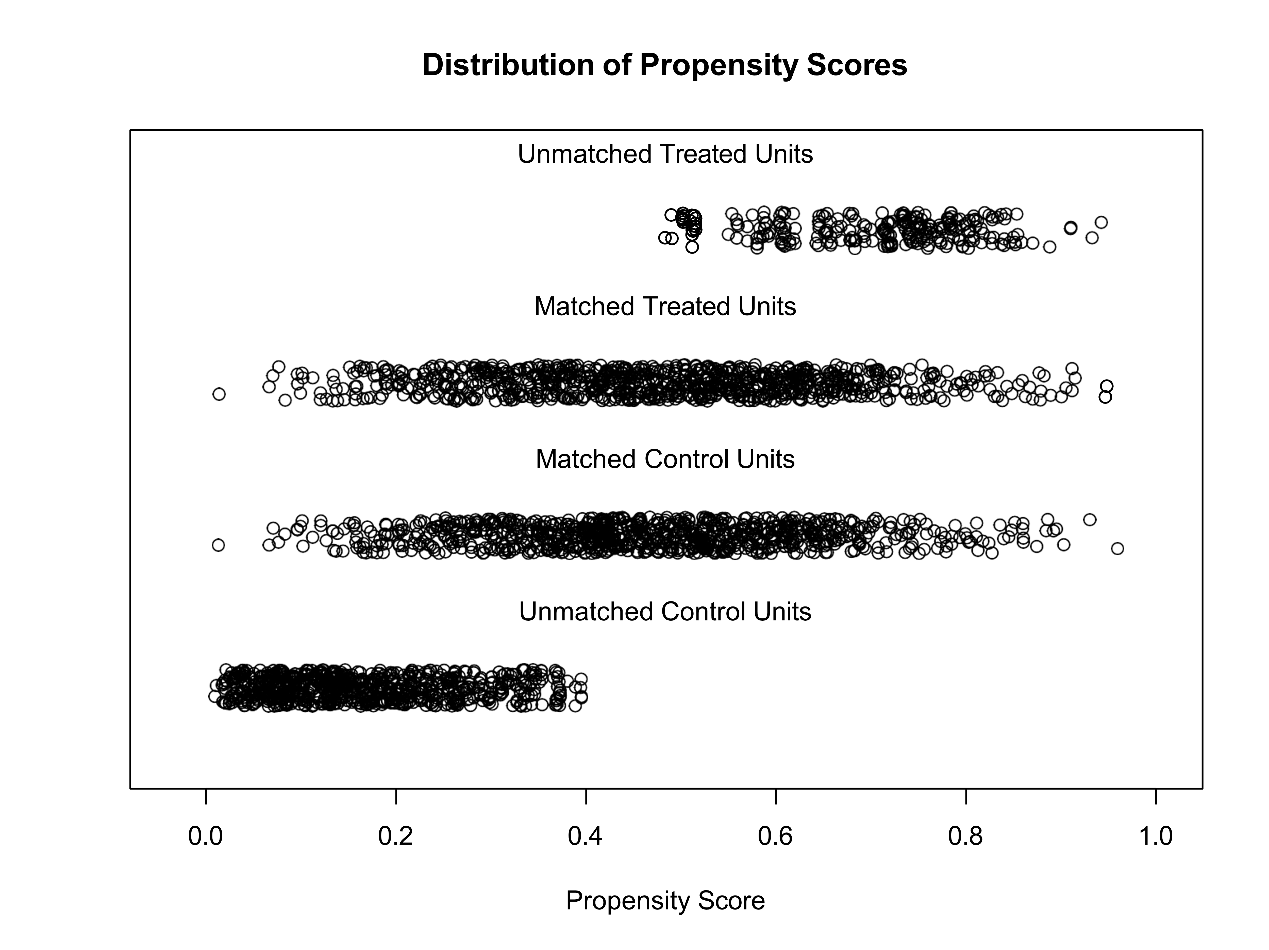


Fig S1 The score distribution of the PSM


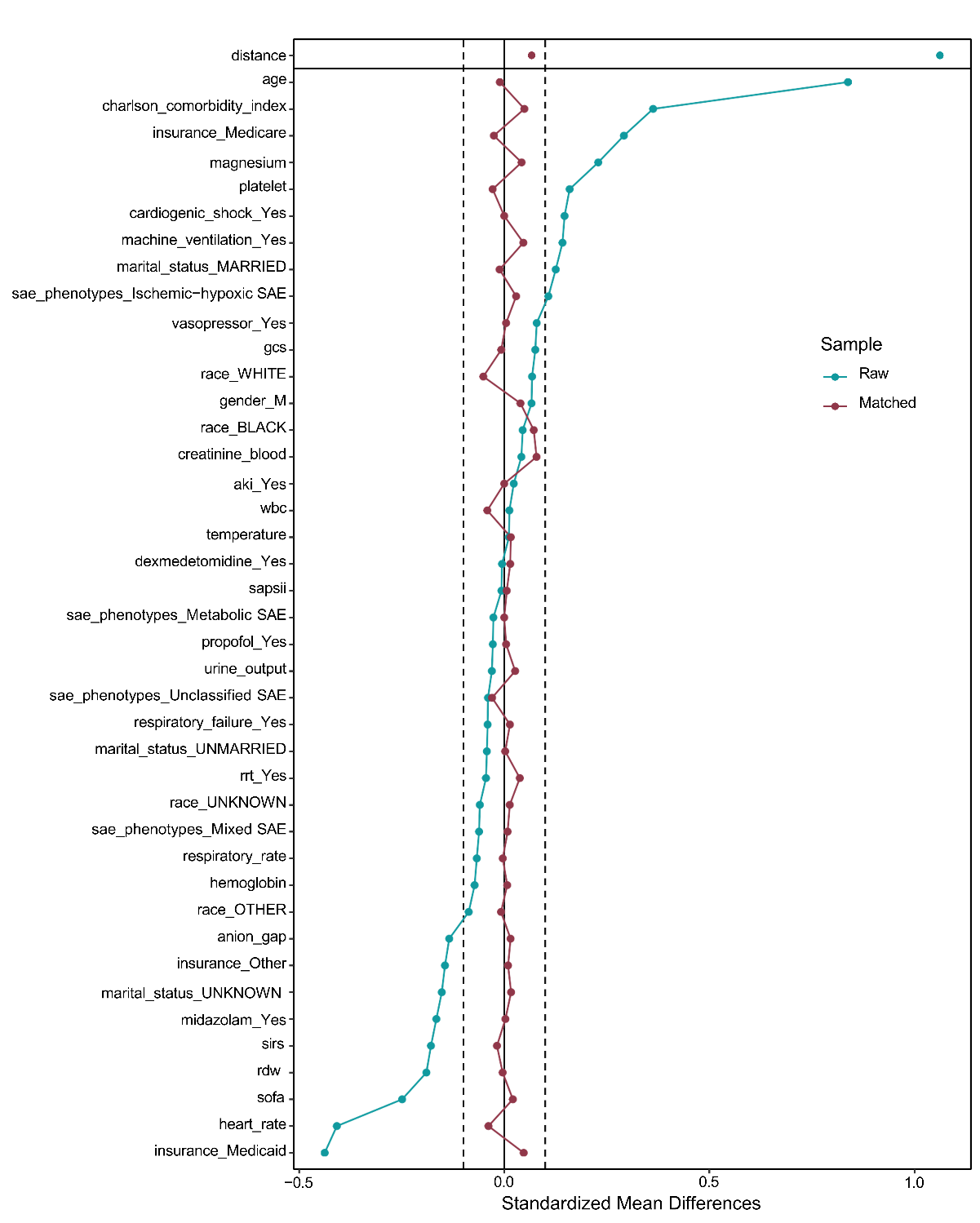


Fig S2 SMD changes of variables before and after matching
